# Teneurin Structure: Splice Variants of a Bacterial Toxin Homolog Specifies Synaptic Connections

**DOI:** 10.3389/fnins.2019.00838

**Published:** 2019-08-07

**Authors:** Demet Araç, Jingxian Li

**Affiliations:** ^1^Department of Biochemistry & Molecular Biology, The University of Chicago, Chicago, IL, United States; ^2^Grossman Institute for Neuroscience, Quantitative Biology and Human Behavior, The University of Chicago, Chicago, IL, United States

**Keywords:** teneurin, ODZ, adhesion GPCR, Latrophilin/ADGRL, synapse, structure, alternative splicing, FLRT

## Abstract

Teneurins are a conserved family of cell-surface adhesion molecules that mediate cellular communication, and play key roles in embryonic and neural development. Their mechanisms of action remained unclear due in part to their unknown structures. In recent years, the structures of teneurins have been reported at atomic resolutions and revealed a clear homology to bacterial Tc toxins with no similarity to other eukaryotic proteins. Another surprising observation was that alternatively spliced variants of teneurins interact with distinct ligands, and thus specify excitatory vs. inhibitory synapses. In this review, we discuss teneurin structures that together with structure-guided biochemical and functional analyses, provide insights for the mechanisms of trans-cellular communication at the synapse and other cell-cell contact sites.

The coupling of extracellular adhesion to intracellular signaling is essential for cells to interpret cues from neighboring cells. With four members in humans, teneurins (TENs) are evolutionarily conserved cell-adhesion proteins that mediate intercellular communication (Baumgartner et al., 1994; Levine et al., 1994; Lossie et al., 2005; Tucker and Chiquet-Ehrismann, 2006; Nakamura et al., 2013; Mosca, 2015). Recent studies show that TENs play central roles in tissue polarity, embryogenesis, heart development, axon guidance, and synapse formation (Levine et al., 1994; Doyle et al., 2006; Silva et al., 2011; Hong et al., 2012; Mosca et al., 2012; Boucard et al., 2014; Mosca and Luo, 2014; Woelfle et al., 2016; Berns et al., 2018). Genetic studies link them to various diseases including neurological disorders, developmental problems, various cancers and congenital general anosmia (Aldahmesh et al., 2012; Ziegler et al., 2012; Hor et al., 2015; Alkelai et al., 2016; Chassaing et al., 2016; Graumann et al., 2017; Talamillo et al., 2017). TENs are type-II transmembrane proteins with large (>2000 amino acids) C-terminal extracellular regions (ECRs) that mediate heterophilic and homophilic trans-cellular interactions that are key for various functions. Most TEN ECRs do not exhibit readily identifiable domains by sequence analysis. Despite the central importance of TENs in multiple physiological functions, the lack of information on the structure of TENs and a molecular understanding of TEN interactions with heterophilic or homophilic ligands has been one of the limiting factors in delineating the mechanisms of TEN action.

## Teneurin Is Homologous to Bacterial Toxins

**FIGURE 1:**
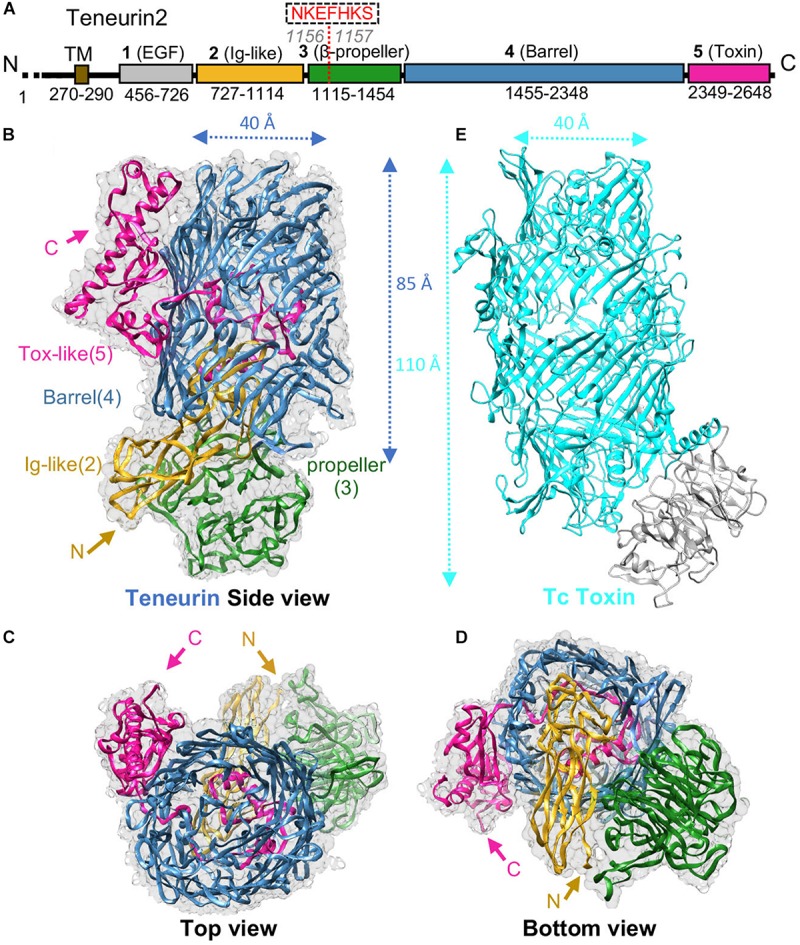
TEN structure reveals homology to bacterial Tc toxins. **(A)** Scheme for human TEN2. Domains are colored gray, yellow, green, blue, and magenta for domains 1-5, respectively; transmembrane region (TM) in colored brown. Residue numbers for each domain are indicated. **(B–D)** Cryo-EM structure of TEN2 ECR (2-5) is shown from different views (Li et al., 2018) (PDB ID: 6CMX). **(E)** The TEN2 domain 4 (blue) is a barrel that is homologous to the BC components of bacterial Tc-toxins (TcC-toxin, cyan, PDB ID: 4O9X). Figure modified from Li et al. (2018).

**FIGURE 2:**
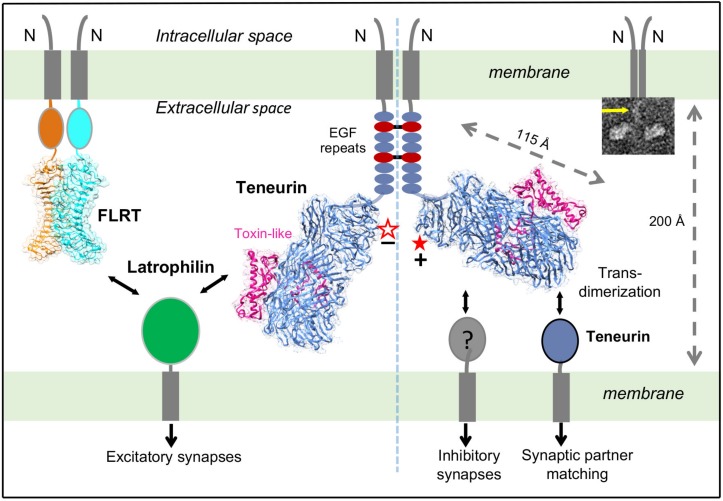
Different splice variants of TENs regulate distinct synapse specifications. Alternative splicing is a molecular switch to regulate which binding partner TEN2 binds to, and what respective function TEN2 does. On the left side, the -SS isoform of TEN2 (empty star) interacts with LPHN. When it is co-expressed with FLRT, TEN2 isoform that lacks the splice insert induces excitatory postsynaptic differentiation. On the right side, TEN2 that includes the splice insert (full star) cannot interact with LPHN, but it can form trans-homodimers to mediate neural circuit-wiring and induces inhibitory synapses. The left and right sides of the TEN2 dimer represent excitatory and inhibitory synapses, respectively. The membranes, teneurin structure (Li et al., 2018) (PDB ID: 6CMX), and distance between the membranes are drawn to scale. Alternative splice site is shown by red star. *Insert:* Representative negative stain EM micrographs of TEN2 ECR(1-5) shows a TEN2 -SS *cis*-dimer. Yellow arrows indicate EGF repeats. Figure modified from Li et al. (2018).

TENs are composed of an N-terminal cytoplasmic tail, a single transmembrane region (TM), and a large ECR (Figure 1A; Baumgartner and Chiquet-Ehrismann, 1993; Baumgartner et al., 1994; Levine et al., 1994; Oohashi et al., 1999). The ECR of TENs comprises eight epidermal growth factor (EGF) motifs that are followed by the large unknown region composed of domains identified as 2, 3, 4 and 5 in Figure 1A. TENs form *cis*-dimers via conserved disulfide bonds formed between their EGF repeats juxtaposed to the transmembrane helix (Oohashi et al., 1999; Feng et al., 2002; Vysokov et al., 2016; Figure 2). Recently, important breakthroughs were achieved and the high resolution cryo-electron microscopy structures of the unknown region from human TEN2, mouse TEN3 and the crystal structure of chicken TEN2 have been reported revealing a highly unusual structure (Jackson et al., 2018; Li et al., 2018). The structures agree with each other and show that the TEN YD repeats corresponding to domain 4 has a striking structural similarity to that of bacterial Tc-toxins as previously suspected (Tucker et al., 2012; Busby et al., 2013; Meusch et al., 2014; Figure 1B). Surprisingly, the TEN2 ECR has an unusual architecture that has not been observed in any other eukaryotic protein before (Figures 1B–D). The structure comprises a large cylindrical β-barrel (blue) sealed at the bottom by an Immunoglobulin (Ig)-like domain (yellow) and a β-propeller (green); and at the top by a C-terminal domain (magenta).

The TEN β-barrel has high homology to bacterial Tc-toxins (Figures 1B,E). Bacterial Tc-toxins are secreted proteins and comprise a large barrel that contains a toxic domain (Landsberg et al., 2011; Busby et al., 2013; Meusch et al., 2014; Figure 1E). The toxin domain is autocleaved from the rest of the protein, passes through the barrel tunnel and is injected into the infected cell to execute its toxic effects usually by binding to the infected cell’s DNA or other components (Figure 1E). The β-barrel of TEN2 structure partially encapsulates a C-terminal toxin-like domain. The toxin-like domain, however, exits the barrel from an opening and is tethered to the outer surface of the barrel (Li et al., 2018; Figure 1B). In spite of structural differences, the autoproteolytic site of the bacterial Tc-toxin is conserved in the TEN2 structure (Li et al., 2018; Arac and Li, 2019) (see Li et al., 2018 and Araç and Li, Current Opinion in Structural Biology, 2019 for in-depth structural analysis and comparison of TEN and bacterial Tc toxin structures). These observations raised numerous questions and exciting possibilities about how TENs may function. An immediate question is whether TENs function similar to bacterial toxins. Is the toxin-like domain of TEN autocleaved, released and inserted into the neighbor cells? Is it toxic to neighbor cells? Does it go into the nucleus and bind to DNA or other components of the cells? Does it mediate intracellular signaling or induce synapse formation and developmental changes? Does the previously reported C-terminal peptide of TEN act like a neuropeptide (Wang et al., 2005; Vysokov et al., 2016)? These are very exciting and open-ended questions that need to be answered in the future.

## Alternative Splicing Regulates Teneurin Interactions

Recent studies provided further surprising results about the roles of splice variants of TENs. TENs are alternatively spliced at two sites within the ECR and include nine- and seven-residue insertions at the EGF repeats and the β-propeller regions, respectively (Tucker et al., 2001). The extracellular region of TENs mediates homophilic and heterophilic interactions that have specific roles in different functions of TENs, however the role of alternative splicing in mediating these interactions remained unknown (Leamey and Sawatari, 2014; Woelfle et al., 2016; Sudhof, 2017). The high-affinity heterophilic interaction of TENs with latrophilins (LPHN1-3), a family of adhesion G Protein Coupled Receptors (GPCRs) that have key roles in synapse development and embryogenesis, regulates synapse formation and organization (Silva et al., 2011; Boucard et al., 2014). On the other hand, trans-homodimerization of TENs is reported to be critical for correct matching of axons with their partner dendrites, a process that is critical for neural circuit-wiring in the nervous system (Hong et al., 2012; Mosca et al., 2012; Mosca, 2015).

Surprisingly, Li et al. (2018) showed that an alternatively spliced seven-residue region (NKEFKHS) within the β-propeller acts as a switch to regulate trans-cellular adhesion of TEN2 to LPHNs (Figure 2, red star). The splice variant that lacks the seven amino acids can bind to full-length LPHN presented on the neighbor cells in cell-aggregation assays and this interaction activates trans-cellular signaling in a LPHN-dependent manner (Figure 2, left side). The other splice variant that includes the seven amino acids, however, is unable to interact with full-length LPHN in identical experiments (Figure 2, right side). Similarly, the same alternatively spliced site that regulates the TEN/LPHN interaction has been reported to also regulate TEN trans-homodimerization (Berns et al., 2018). Intriguingly, the splice variant that lacks these seven amino acids cannot mediate trans-homodimerization, whereas the other variant can (Figure 2, right side). Taken together, these results suggest that TEN splice variants can either interact with LPHNs or mediate trans-dimerization with itself, but a single variant cannot mediate both interactions.

## Different Splice Variants of Tens Perform Distinct Synapse Specifications

As different TEN splice variants are involved in different ligand interactions, the next question arose: Do different TEN splice variants perform biologically distinct functions? Subsequent studies by Li et al. (2018) examined the capability of TEN2 + SS and TEN2 -SS to induce artificial synapse formation. In these assays, HEK293 cells expressing TEN variants were co-cultured with primary neurons; and inhibitory and excitatory synapse formation was monitored for pre- and post-synaptic differentiation for both types of synapses (Li et al., 2018). The results showed that TEN2 + SS induced GABAergic (inhibitory) synaptic specializations but failed to induce glutamatergic (excitatory) synaptic specifications (Figure 2, right side; Li et al., 2018).

On the other hand, initially, TEN2 -SS failed to recruit both excitatory and inhibitory synaptic markers (Li et al., 2018). However, when fibronectin leucine rich repeat transmembrane protein (FLRT3), another LPHN ligand that on its own is unable to induce pre- or postsynaptic specializations, was coexpressed in HEK293T cells with the TEN2 -SS, these molecules together potently induced excitatory but not inhibitory postsynaptic specializations (Sando et al., 2019; Figure 2, left side). The other splice variant, TEN + SS (that cannot bind LPHN and cannot induce excitatory synapses) was still inactive in excitatory synapse formation, even when coexpressed with FLRT (Sando et al., 2019). Thus, TEN -SS and FLRT can stimulate excitatory synapse formation in combination, but not separately. These results are consistent with studies in transgenic LPHN mice *in vivo*, where coincident binding of both TEN and FLRT to LPHN is required for excitatory synapse formation (Sando et al., 2019).

As we start to understand the functions of TENs, these observations are likely only a glimpse of the complexity of the TEN system. Many questions arise: How can a seven-residue splice site affect ligand binding that is away from the ligand binding site on TEN (Figure 2, left side; Li et al., 2018). What are the implications of various TEN variants and isoforms for neural wiring? Do other splice variants have important biological functions? What are the other unknown ligands for TENs? What is the role of TEN/LPHN interaction in other systems such as embryonic development? TEN field is awaiting further exciting and surprising findings.

## Author Contributions

Both authors wrote the manuscript.

## Conflict of Interest Statement

The authors declare that the research was conducted in the absence of any commercial or financial relationships that could be construed as a potential conflict of interest.
